# LINE-2 transposable elements are a source of functional human microRNAs and target sites

**DOI:** 10.1371/journal.pgen.1008036

**Published:** 2019-03-13

**Authors:** Rebecca Petri, Per Ludvik Brattås, Yogita Sharma, Marie E. Jönsson, Karolina Pircs, Johan Bengzon, Johan Jakobsson

**Affiliations:** 1 Laboratory of Molecular Neurogenetics, Department of Experimental Medical Science, Wallenberg Neuroscience Center and Lund Stem Cell Center, Lund University, Lund, Sweden; 2 Department of Clinical Sciences, Division of Neurosurgery, Lund Stem Cell Center, Lund University and Region Skåne, Lund, Sweden; Cornell University, UNITED STATES

## Abstract

Transposable elements (TEs) are dynamically expressed at high levels in multiple human tissues, but the function of TE-derived transcripts remains largely unknown. In this study, we identify numerous TE-derived microRNAs (miRNAs) by conducting Argonaute2 RNA immunoprecipitation followed by small RNA sequencing (AGO2 RIP-seq) on human brain tissue. Many of these miRNAs originated from LINE-2 (L2) elements, which entered the human genome around 100–300 million years ago. L2-miRNAs derived from the 3’ end of the L2 consensus sequence and thus shared very similar sequences, indicating that L2-miRNAs could target transcripts with L2s in their 3’UTR. In line with this, many protein-coding genes carried fragments of L2-derived sequences in their 3’UTR: these sequences served as target sites for L2-miRNAs. L2-miRNAs and their targets were generally ubiquitously expressed at low levels in multiple human tissues, suggesting a role for this network in buffering transcriptional levels of housekeeping genes. In addition, we also found evidence that this network is perturbed in glioblastoma. In summary, our findings uncover a TE-based post-transcriptional network that shapes transcriptional regulation in human cells.

## Author summary

Transposable elements (TEs) are repetitive sequences, that have contributed to the landscaping of the genome by jumping into new positions and amplifying in number. TEs have been suggested to play a role in gene regulation, but it remains poorly understood how they contribute to this process. In this study, we show that in various human tissues, an ancient class of TEs give rise to small non-coding RNAs, called microRNAs (miRNAs), that are important regulators of gene expression. The same class of TEs also serves as target sites for these TE-derived miRNAs when they are part of protein-coding transcripts. We also provide evidence that TE-derived miRNAs and target sites may play a role in human disease, as they are dysregulated in aggressive brain tumors. Altogether, our study provides novel insight into how TEs acting as miRNAs play a role in gene regulation in both, healthy and diseased human tissues.

## Introduction

The emergence and evolution of gene regulatory networks is thought to underlie biological adaptations and speciation. Transposable elements (TEs) have been implicated in these processes because they can amplify in numbers and move into new regions of the genome. Genomic analyses indicate that TEs have a role in gene regulatory networks, since a substantial fraction of TEs evolve under selective constraints despite being non-coding [1]. However, the actual impact of TEs on human transcriptional networks remains poorly understood.

We and others have recently described that many transcripts expressed in various human tissues contain TE-derived sequences [2, 3]. Many of these sequences appear to be indirectly transcribed, often in antisense direction, as part of other transcripts, including those coding for protein [3]. Together with the finding that TEs can generate miRNA target sites in the 3’UTR of protein-coding transcripts [4–8], the data indicate that TEs within transcripts can, at least in part, act as templates for RNA-binding proteins such as the microRNA (miRNA) machinery, and contribute to post-transcriptional regulation.

In addition, TEs can be a source of non-coding RNAs, such as long non-coding RNAs (lncRNAs) or miRNAs. Computational and experimental studies have shown that LINE (e.g. LINE-1, LINE-2, LINE-3), SINE (e.g. MIR and Alu), and some LTR transposons, can act as miRNA sources [8–10]. miR-28, miR-95, and miR-151 are examples of functionally validated miRNAs that are derived from LINE-2 (L2) sequences [6, 11, 12]. However, current studies demonstrating functional TE-derived miRNAs and target sites are mainly conducted in cell lines and functional evidence for a TE-based post-transcriptional network in primary tissues, such as the human brain, is missing.

In this study, we used Argonaute2 RNA immunoprecipitation (AGO2 RIP) on adult human brain tissue. We found that many small RNAs bound by AGO2 are derived from TEs, with the majority being derived from L2 elements, which have previously been annotated as miRNAs. These L2-miRNAs show strong sequence complementarity to L2 elements found in the 3’UTR of protein-coding genes. Transcripts containing L2 elements in the 3’UTR are incorporated into the RNA-induced silencing complex (RISC) in the human brain and are regulated by L2-miRNAs. We found that L2-miRNAs and targets are generally ubiquitously expressed in multiple human tissues, hereby primarily acting on housekeeping genes. We also found that this L2-miRNA network is perturbed in samples from patients with glioblastoma. Altogether, our results demonstrate a TE-based post-transcriptional network that influences the expression of protein-coding genes in human tissues.

## Results

### Identification of transposable element-derived microRNAs expressed in the human brain

**Fig 1:**
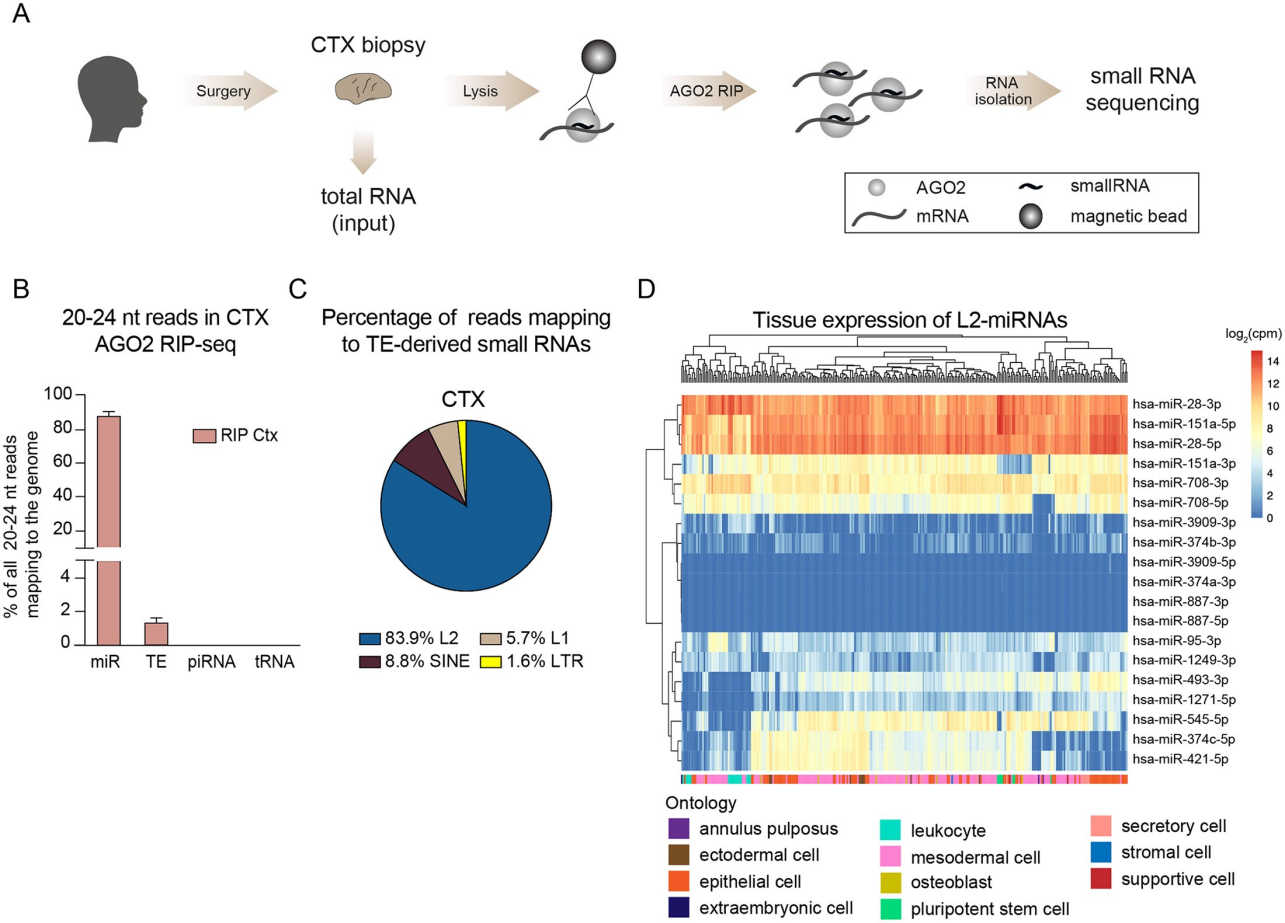
AGO2-associated small RNAs in human cortex tissue. A) Schematics of AGO2 RIP-seq on human cortex samples followed by small RNA sequencing. B) Bar graph showing the percentage of reads of 20–24 nucleotides (nt) in the human genome mapping to mature miRNAs (miR), transposable elements (TE), piwi RNAs (piRNA), and transfer RNAs (tRNA). Data are represented as mean ± SEM (RIP Ctx n = 3). C) Pie chart showing the percentage of reads mapping to TEs. D) Heatmap depicting the expression of L2-miRNAs (log_2_(cpm)) across different human tissues and cell types (n = 400). AGO2—Argonaute2, AGO2 RIP—AGO2 RNA immunoprecipitation, CTX—cortex, L1—LINE-1, L2—LINE-2, LTR—long terminal repeat, mir—precursor miRNA, SINE—short interspersed nuclear element.

To identify small RNAs that participate in gene silencing in the human brain we performed Argonaute2 RNA immunoprecipitation followed by small RNA sequencing (AGO2 RIP-seq) on surgical biopsies obtained from the human cortex (n = 3) (Fig 1A). The use of AGO2 RIP-seq on fresh human brain tissue circumvents several challenges associated with detecting functional small RNAs derived from transposable elements (TEs): it reduces background noise generated by degradation products, as well as avoiding problems arising from the use of cell lines, where a loss of DNA methylation could activate aberrant TE expression.

We found that AGO2-bound small RNAs displayed a high enrichment for reads of 20–24 nucleotides (nt), the typical size of microRNAs (miRNAs), while input samples, which include all small RNAs in the tissue, displayed an expected broad size profile of RNAs including, for example, many RNAs in the size range of 30–36 nt (S1A Fig). We next investigated the genomic origin of small RNAs expressed in the human brain. As expected, most AGO2-bound RNAs were classical miRNAs (S1B Fig); we found very limited evidence for AGO2-binding of transfer RNA fragments (tRFs) or piwi-RNAs (piRNA), although tRFs, mostly 30–36 nt in size, were abundant in input samples (S1B Fig).

When focusing our analyses on AGO2-bound small RNAs of 20–24 nt, we detected high expression of classic brain-enriched miRNAs, such as miR-128 (S1C Fig) [13]. Interestingly, we also found a substantial fraction (around 2–3% of reads) of AGO2-bound RNAs that mapped to TEs, including LINE, LTR, and SINE elements (Fig 1B).

### L2-microRNAs are abundantly expressed in human tissues

To identify miRNA-like small RNAs that are derived from TEs, we aligned reads with a length of 20–24 nt to the human reference genome (hg38) and removed all reads that mapped equally well to more than one locus (see Methods for a more detailed description). Surprisingly, the great majority (83.9%) of the identified TE-derived small RNAs originated from L2 elements (Fig 1C). When comparing the L2-derived small RNAs with miRbase annotations, we found that they have previously been identified as miRNAs: these include, for example, L2c-derived miR-151 and L2b-derived miR-95 (Table 1) [9, 14].

**Table 1:** L2-derived miRNAs expressed in human brain tissue.

TE	coordinates	direction of miRNA	miRNA	paired	mean expression (reads in RIP)
L2b	chr 4: 8005199–8005343 (+)	AS	miR-95-3p	L2c	8344.66
L2c	chr 8: 140732529–140732650 (-)	S	miR-151a-3p/-5p	L2c	5253.32
L2c	chr 8: 140732622–140732734 (+)	AS	miR-151a-5p	L2c	29.13
L2c	chr 3: 188688700–188688813 (-)	AS	miR-28-5p	L2c	771.25
L2c	chr 3: 188688783–188688877 (+)	S	miR-28-3p/-5p	L2c	299.29
L2c	chr 11: 79402022–79402117 (+)	AS	miR-708-3p/-5p	L2c	695.05
L2c	chr 11: 79401986–79402051 (-)	S	miR-708-3p	L2c	217.33
L2c	chr 22: 45200672–45200995 (-)	S	miR-1249-3p	-	443.35
L2c	chr X: 74287157–74287324 (-)	S	miR-374a-3p/ miR-545-5p	-	90.09
L2c	chr X: 74218419–74218583 (-)	S	miR-374b-3p/ miR-421-5p	-	12.58
L2c	chr X: 74218419–74218583 (-)	AS	miR-374c-5p	-	12.58
L2b	chr14: 100869090–100869150 (-)	S	miR-493-3p	-	49.70
L2b	chr 5: 176367379–176367999 (+)	S	miR-1271-5p	L2a	31.11
L2c	chr 22: 35335672–35335822 (-)	AS	miR-3909-3p/-5p	-	71.28
L2a	chr 5: 15934793–15935362 (-)	AS	miR-887-3p/-5p	-	9.34

To investigate the similarity between different L2-miRNAs, we focused on those derived from the L2c-subfamily. We mapped all L2c-miRNAs detected to the L2c consensus sequence (RepeatMasker). This analysis showed that many L2c-miRNAs are similar in sequence, since they are generated from the same position within the 3’ end of the L2c consensus, differing by only a few bases and therefore most likely sharing many targets (S1D Fig).

By using our strict alignment strategy, we detected in total 19 miRNAs that were derived from L2 sequences and that were expressed in the human brain (Table 1, Fig 1D). To investigate the expression of these L2-miRNAs in other human tissues we used a publicly available small RNA-seq dataset from 400 human samples [15]. We found that most L2-miRNAs are ubiquitously expressed, albeit at different levels, including high-level expression of miR-28-3p, miR-28-5p, and miR-151a-5p (Fig 1D). In contrast, we found very limited evidence for cell-type-specific expression of L2-miRNAs (Fig 1D).

### L2-derived miRNAs are expressed in the mouse brain

**Fig 2:**
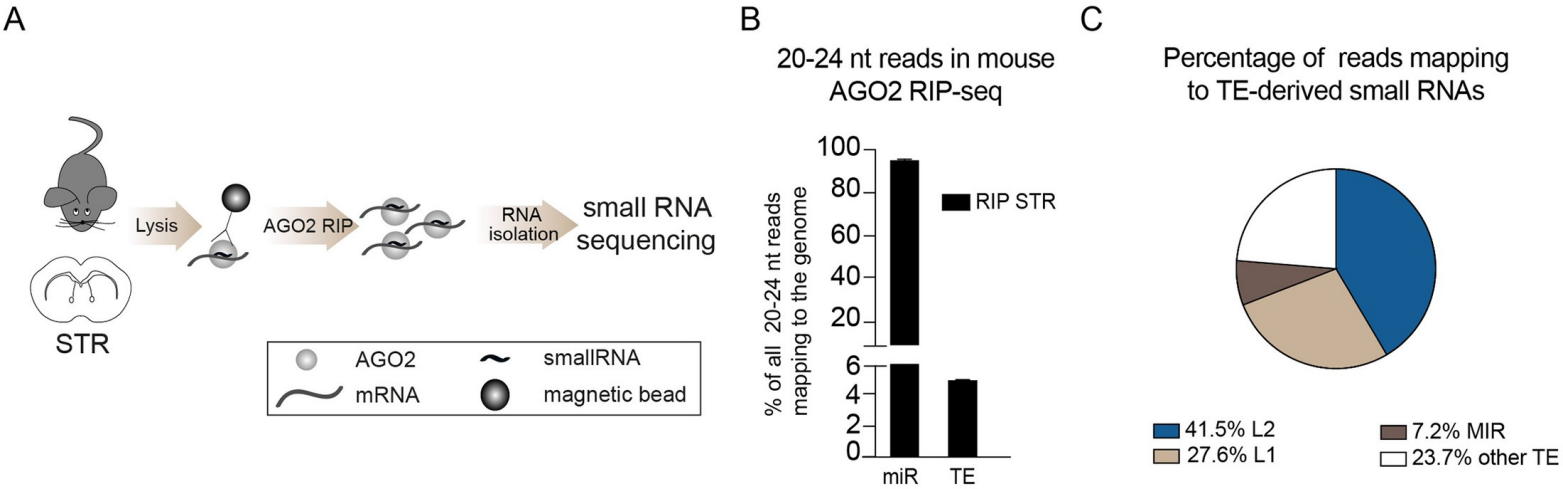
AGO2-associated small RNAs in the mouse brain. A) Schematics of AGO2 RIP-seq on mouse striatum (STR) followed by small RNA sequencing. B) Bar graph showing the percentage of reads of 20–24 nt in the mouse genome mapping to mature miRNAs (miR) and transposable elements (TE). Data are represented as mean ± SEM (RIP STR n = 3). C) Pie chart showing the percentage of reads mapping to TEs.

To investigate whether L2-miRNAs are conserved among mammals, we also conducted AGO2 RIP-seq on mouse brain tissue (Fig 2A). We found a similar proportion of TE-derived miRNAs in the mouse brain as in the human brain, with L2-miRNAs being the most common TE-derived miRNAs also found in mouse (Fig 2B and 2C, Table 2).

**Table 2:** L2-derived miRNAs expressed in mouse brain tissue.

TE	coordinates	direction of miRNA	miRNA	paired	mean expression (reads in RIP)
L2c	chr 15: 73254797–73254856 (-)	S	miR-151-3p/-5p	L2c	41322
L2c	chr 15: 73254854–73254920 (+)	AS	miR-151-5p	L2c	6
L2c	chr 7: 96249497–96249546 (+)	S	miR-708-3p	L2a	25417
L2a	chr 7: 96249441–96249478 (-)	AS	miR-708-5p	L2c	183
L2c	chr 4: 94665178–94665336 (-)	S	miR-872-3p/-5p	-	2965
L2c	chr 15: 84951302–84951582 (-)	S	miR-1249-3p	-	2064
L2c	chr 16: 24827889–24827951 (+)	S	miR-28a-3p	L2c	392
L2c	chr 16: 24827826–24827887 (-)	AS	miR-28a-5p	L2c	65
L2c	chr X: 103572919–103572951 (+)	AS	miR-421-3p	L2c	284
L2c	chr X: 103572957–103573111 (-)	S	miR-421-5p/ miR-374b-3p	L2c	37
L2c	chr X: 103572957–103573111 (-)	AS	miR-374c-5p	L2c	37
L2b	chr 12: 109580258–109580318 (-)	AS	miR-493-3p/-5p	-	161
L2a	chr X: 105379137–105379236 (-)	S	miR-325-5p	L2b	110
L2b	chr X: 105378963–105379128 (+)	AS	miR-325-3p	L2a	40
L2	chr 10: 128382803–128382885 (-)	AS	miR-6914-3p/-5p	-	41
L2a	chr X:147010598–147010647 (+)	S	miR-1264-5p	-	29

Several of the L2-miRNAs were conserved in sequence and genomic location between mouse and human including, for example, miR-151 and miR-28 (Table 3). However, we also noted that some L2-miRNAs were present in the human but not in the mouse genome, e.g. miR-95 (Table 3). This shows that L2-miRNAs are bound by AGO2 and expressed in the brain of different mammalian species, suggesting a conserved functional role for these non-coding RNAs. However, it is also worth noting that we detected a higher proportion of miRNAs derived from other TEs such as LINE-1 (L1) and MIRs in the mouse brain compared to human samples.

**Table 3:** Conservation of L2-derived miRNAs.

human L2-microRNA (expressed in brain)	orthologue mouse L2-microRNA	sequence comparison		conserved in genomic location
hsa-miR-95-5p	-	hsa-miR-95-5p	UCAAUAAAUGUCUGUUGAAUU	-
hsa-miR-95-3p	-	hsa-miR-95-3p	UUCAACGGGUAUUUAUUGAGCA	-
hsa-miR-151a-5p	mmu-miR-151-5p	hsa-miR-151a-5p	UCGAGGAGCUCACAGUCUAGU	yes
		mmu-miR-151-5p	UCGAGGAGCUCACAGUCUAGU	
hsa-miR-151a-3p	mmu-miR-151-3p	hsa-miR-151a-3p	CUAGACUGAAGCUCCUUGAGG	yes
		mmu-miR-151-3p	CUAGACUGAGGCUCCUUGAGG	
hsa-miR-28-5p	mmu-miR-28a-5p	hsa-miR-28-5p	AAGGAGCUCACAGUCUAUUGAG	yes
		mmu-miR-28a-5p	AAGGAGCUCACAGUCUAUUGAG	
hsa-miR-28-3p	mmu-miR-28a-3p	hsa-miR-28-3p	CACUAGAUUGUGAGCUCCUGGA	-
		mmu-miR-28a-3p	CACUAGAUUGUGAGCUGCUGGA	
mmu-miR-708-3p	mmu-miR-708-3p	hsa-miR-708-3p	CAACUAGACUGUGAGCUUCUAG	yes
		mmu-miR-708-3p	CAACUAGACUGUGAGCUUCUAG	
hsa-miR-708-5p	mmu-miR-708-5p	hsa-miR-708-5p	AAGGAGCUUACAAUCUAGCUGGG	yes
		mmu-miR-708-5p	AAGGAGCUUACAAUCUAGCUGGG	
hsa-miR-1249-3p	mmu-miR-1249-3p	hsa-miR-1249-3p	ACGCCCUUCCCCCCCUUCUUCA	yes
		mmu-miR-1249-3p	ACGCCCUUCCCCCCCUUCUUCA	
hsa-miR-1249-5p	mmu-miR-1249-5p	hsa-miR-1249-5p	AGGAGGGAGGAGAUGGGCCAAGUU .	yes
		mmu-miR-1249-5p	AGGAGGGAGGGGAUGGGCCAAGUUC	
hsa-miR-374a-3p	-	hsa-miR-374a-3p	CUUAUCAGAUUGUAUUGUAAUU	-
hsa-miR-374a-5p	-	hsa-miR-374a-5p	UUAUAAUACAACCUGAUAAGUG	-
hsa-miR-374b-3p	mmu-miR-374b-3p	hsa-miR-374b-3p	CUUAGCAGGUUGUAUUAUCAUU	yes
		mmu-miR-374b-3p	GGUUGUAUUAUCAUUGUCCGAG	
hsa-miR-374b-5p	mmu-miR-374b-5p	hsa-miR-374b-5p	AUAUAAUACAACCUGCUAAGUG	yes
		mmu-miR-374b-5p	AUAUAAUACAACCUGCUAAGUG	
hsa-miR-374c	mmu-miR-374c-5p	hsa-miR-374c-5p	AUAAUACAACCUGCUAAGUGCU	yes
		mmu-miR-374c-5p	AUAAUACAACCUGCUAAGUG‥	
hsa-miR-493-3p	mmu-miR-493-3p	hsa-miR-493-3p	UGAAGGUC . UACUGUGUGCCAGG	yes
		mmu-miR-493-3p	UGAAGGUCCUACUGUGUGCCAGG	
hsa-miR-1271-5p	-	hsa-miR-1271-5p	CUUGGCACCUAGCAAGCACUCA	-
hsa-miR-3909-3p	-	hsa-miR-3909-3p	CAGCAGGCUCCGGGGGACAGGG	-
hsa-miR-3909-5p	-	hsa-miR-3909-5p	UGUCCUCUAGGGCCUGCAGUCU	-
hsa-miR-887-3p	-	hsa-miR-887-3p	GUGAACGGGCGCCAUCCCGAGG	-

### Biogenesis of L2-derived miRNAs

**Fig 3:**
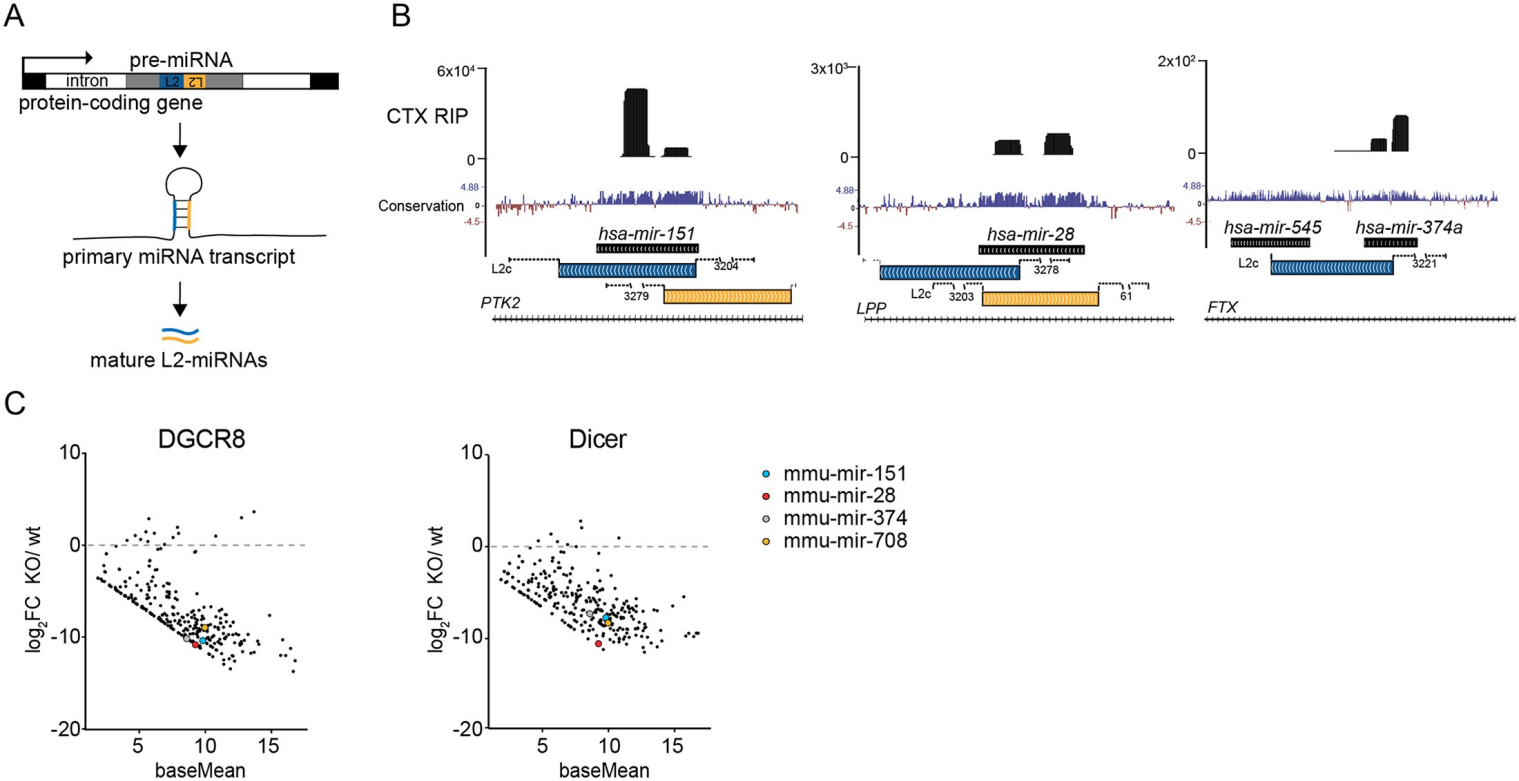
Biogenesis of L2-derived miRNAs. A) Schematics of transcription and biogenesis of L2-derived miRNAs. B) UCSC genome browser tracks showing examples of L2-miRNAs in AGO2 RIP-seq samples from human brain tissue. C) Dot plots showing miRNA hairpin levels in DGCR8 and Dicer knockout mESCs [16]. The base mean (x-axis) is plotted against the log_2_-transformed fold change of knockout versus wild-type samples (y-axis).

Most L2 elements in the human genome are 5’truncated and thereby lack their own promoter. To investigate how L2-derived miRNAs are transcribed we analyzed publicly available Fantom5 CAGE data [15] to identify the transcription start site (TSS) of pri-miRNA transcripts. We found that all human L2-miRNAs expressed in our data set were located within introns of protein-coding genes or long non-coding (lncRNAs) exploiting their promoters to drive transcription of the pri-miRNA transcripts that contain L2 fragments (Fig 3A and S2A Fig).

We also noted that many of the L2-miRNAs originate from two distinct L2 elements that are located close to each other and oriented in opposite directions, thereby providing a source of hairpin structures (Fig 3B), as previously described [14]. However, we also found cases where a pri-miRNA transcript with a single L2 element gives rise to two miRNA precursors e.g. *mir-545* and *mir-374* (Fig 3B).

In order to investigate if L2-miRNAs are processed through the canonical miRNA-biogenesis pathway we analyzed previously published small RNA-seq data from Dicer-KO and DGCR8-KO mouse embryonic stem cells [16]. We found that L2-miRNAs depend on both DGCR8 and Dicer, confirming that maturation of these miRNAs follow the canonical miRNA biogenesis pathway (Fig 3C).

Together, these data demonstrate that L2-miRNAs are products of pri-miRNAs originating from introns of protein-coding transcripts or lncRNAs and that they are processed through the canonical miRNA-pathway.

### L2-derived fragments are found in the 3’UTR of protein-coding genes

L2-derived AGO2-associated miRNAs have a large number of potential “self-targets” since these elements extensively colonized the genome of our ancestors around 100–300 million years ago, resulting in almost 500,000 L2-derived fragments in the human genome [17]. Thus, L2-miRNAs could potentially guide the RISC to 3’UTRs containing these elements transcribed in the opposite direction of their element of origin, thereby providing a possibility for TEs to post-transcriptionally influence the expression of numerous protein-coding genes. To investigate this possibility, we analyzed the location of L2 elements in the human genome and found that a substantial number of L2 elements, 2847, are located in 3’UTRs. They could therefore act as miRNA targets.

**Fig 4:**
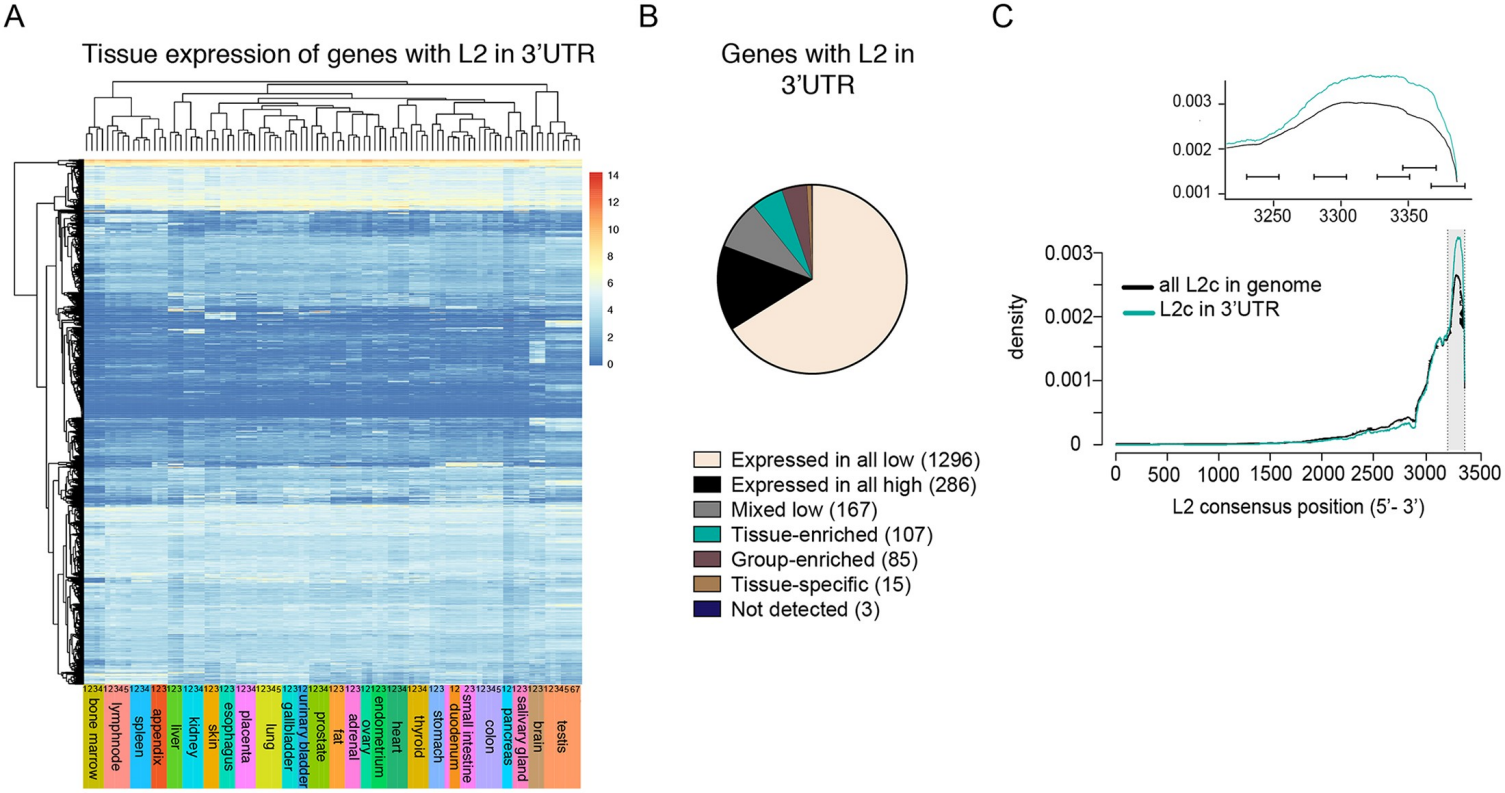
Expression of L2-carrying genes in human tissues. A) Heatmap depicting the expression of L2-carrying genes across 27 tissue samples obtained from 95 individuals [18]. B) Pie chart showing the number of expressed genes classified based on the Fagerberg tissue-specificity classification [18]. C) Graph showing the density of the L2c consensus sequence of all L2c elements in the genome (black line) and of L2c elements in the 3’UTR (blue line).

To investigate the expression profile of these genes in various human tissues we used a publicly available data set containing RNA-seq from 27 different human tissues and cells [18]. We found that the majority of genes that carry L2s in the 3’UTR were ubiquitously expressed (Fig 4A). Using the classification for tissue-specific expression from Fagerberg et al. [18], we found that the vast majority of these genes were expressed in all tissues at low levels (Fig 4B). In contrast, only a small proportion of these genes displayed tissue-specific expression (Fig 4B).

We next analyzed the structural conservation of L2 elements in the human genome, focusing on the L2c family. We found that the majority of L2c are 5’-truncated, as is often the case for LINE elements. Interestingly, when analyzing L2c elements located in the 3’UTR of protein-coding genes specifically, we found an even stronger conservation of the very 3’-end of the L2 element, which corresponds to the region which generates the L2-miRNAs (Fig 4C). Together, these data demonstrate that around 2,000 genes, mostly housekeeping genes expressed at low levels, carry an L2 element in the 3’UTR. These L2 elements have the potential to act as target sites for the RISC, since they are conserved to match the sequence of the L2-miRNAs.

### L2-derived fragments in the 3’UTR of protein-coding genes are L2-microRNA-targets

**Fig 5:**
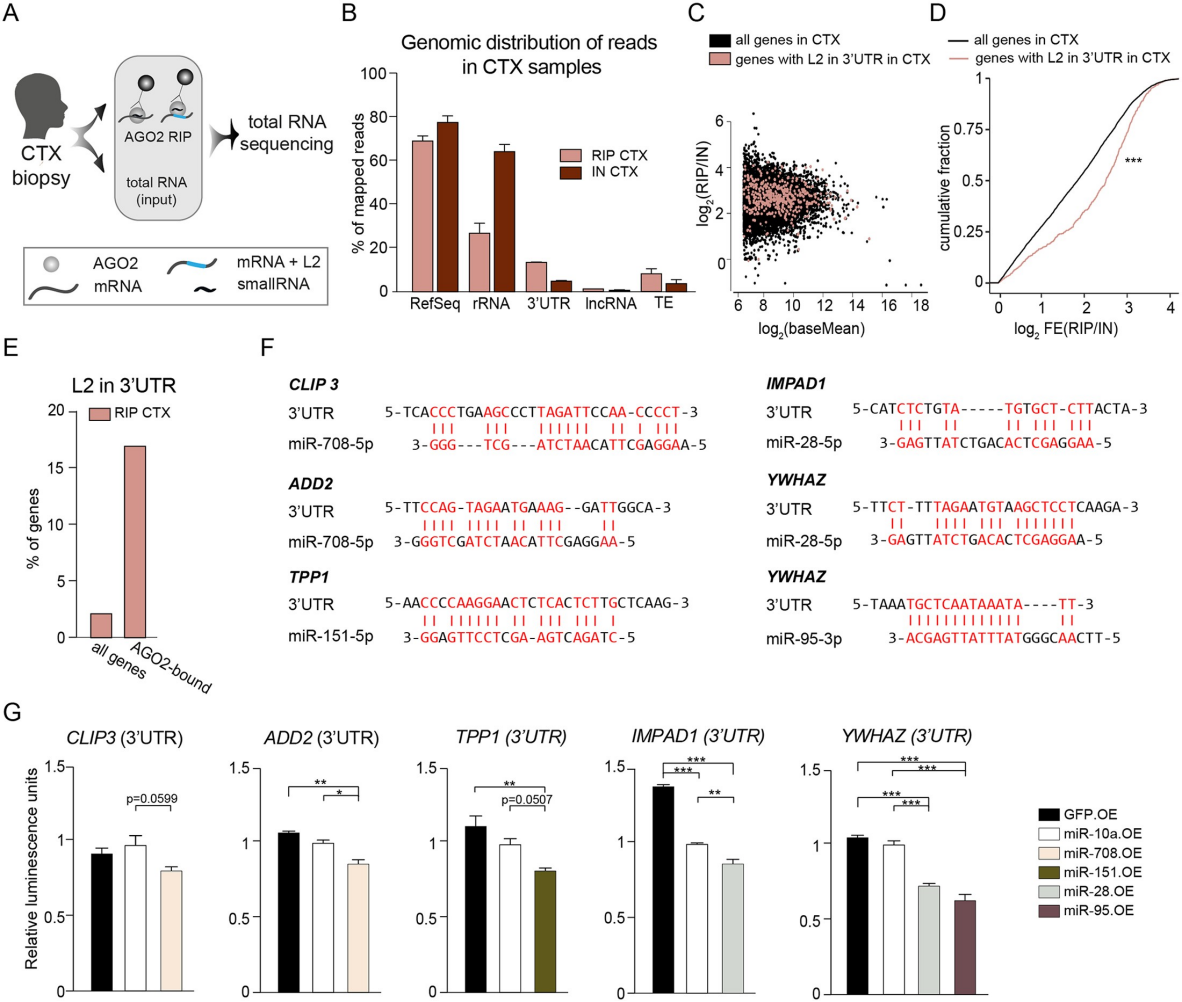
Genes with L2 in 3’UTR are bound by AGO2 in the human brain. A) Schematics of AGO2 RIP followed by total RNA sequencing on cortex (CTX) biopsies. B) Genomic distribution of RNAs in the AGO2 RIP-seq and input samples of cortex (CTX, n = 2). C) Dot plot of all genes in cortex samples from the human brain. Genes with L2 in the 3’UTR are marked in red. The log_2_-transformed mean expression (x-axis) is plotted against the log_2_-transformed fold change of RIP (n = 2) versus input samples (n = 2) (y-axis). D) Cumulative fraction plot of the fold changes of all genes and genes with L2 in their 3’UTR in RIP versus INPUT samples. ***p<0.001 Kolmogorov–Smirnov test. E) Bar plot showing the percentage of genes within the 3’UTR of all genes, and of the top 100 highest-expressed genes that are more than 4-fold enriched in RIP compared to input samples. F) Potential target sites of L2-miRNAs in the L2 sequence in 3’UTRs of genes. Complementary bases are marked in red and with a vertical line. G) Luciferase assay of L2-derived target sites. Data are shown as mean ± SEM. *p<0.05, **p<0.01, ***p<0.001, one-way ANOVA followed by a Tukey’s multiple comparison post-hoc test. AGO2—Argonaute2, AGO2 RIP—AGO2 RNA immunoprecipitation, CTX—cortex, L2—LINE-2, lncRNA—long non-coding RNA, OE—overexpressor, TE—transposable elements, rRNA—ribosomal RNA.

To provide functional evidence that L2-containing 3’UTRs are regulated by miRNAs in the human brain, we conducted AGO2 RIP on cortex tissue (n = 2), followed by total RNA sequencing to identify miRNA target genes (Fig 5A). As expected, most reads in the RIP samples mapped to RefSeq annotations (Fig 5B), while input samples were rich in ribosomal RNA (rRNA). The amount of rRNA was, as expected, strongly decreased in the RIP samples (Fig 5B). We found an enrichment of reads mapping to 3’UTRs of genes in the RIP samples compared to input fractions, which is in line with the high prevalence of miRNA target sites in this part of a transcript (Fig 5B). Strikingly, we also found that reads mapping to TEs were enriched in RIP samples.

We next analyzed all genes with L2 elements in their 3’UTR (Fig 5C). We assessed the global distribution of transcripts bound to AGO2 using a cumulative fraction graph and found that genes with L2 elements in the 3’UTR were significantly enriched in AGO2-binding compared to all genes (Fig 5D). We next set stringent criteria for identifying high-confidence AGO2-bound genes. We selected the 100 most abundant transcripts in the RIP samples which were also more than 4-fold enriched in RIP compared to the input fractions. Strikingly, we found that among the AGO2-bound genes, L2-carrying transcripts were highly enriched and made up 15% of the transcripts (Fig 5E). In comparison, approximately 2% of all transcripts detected in this analysis carry L2 in their 3’UTR. These analyses show that genes carrying L2 in their 3’UTR are present and enriched in the AGO2 RIP fractions of human brain tissue samples, suggesting that they are functional miRNA target genes.

When we searched for potential miRNA target sites within the L2 elements in the 3’UTRs of AGO2-bound genes, we found several potential non-canonical target sites which are highly complementary to the L2-miRNAs: miR-28, miR-95, miR-151a, and miR-708 (Fig 5E). To validate the functionality of these non-canonical target sites, we performed luciferase assays for 6 L2-target sites and confirmed that all tested target sites were regulated by L2-miRNAs (Fig 5F). It is worth noting that out of the 6 validated target sites, one is completely conserved in mouse while one target has only a single nucleotide change, suggesting that parts of this networks are conserved. Taken together, these data provide functional evidence demonstrating that L2-miRNAs regulate target genes carrying L2-derived sequences in their 3’UTR.

### Dysregulation of L2-microRNAs in human glioblastoma

**Fig 6:**
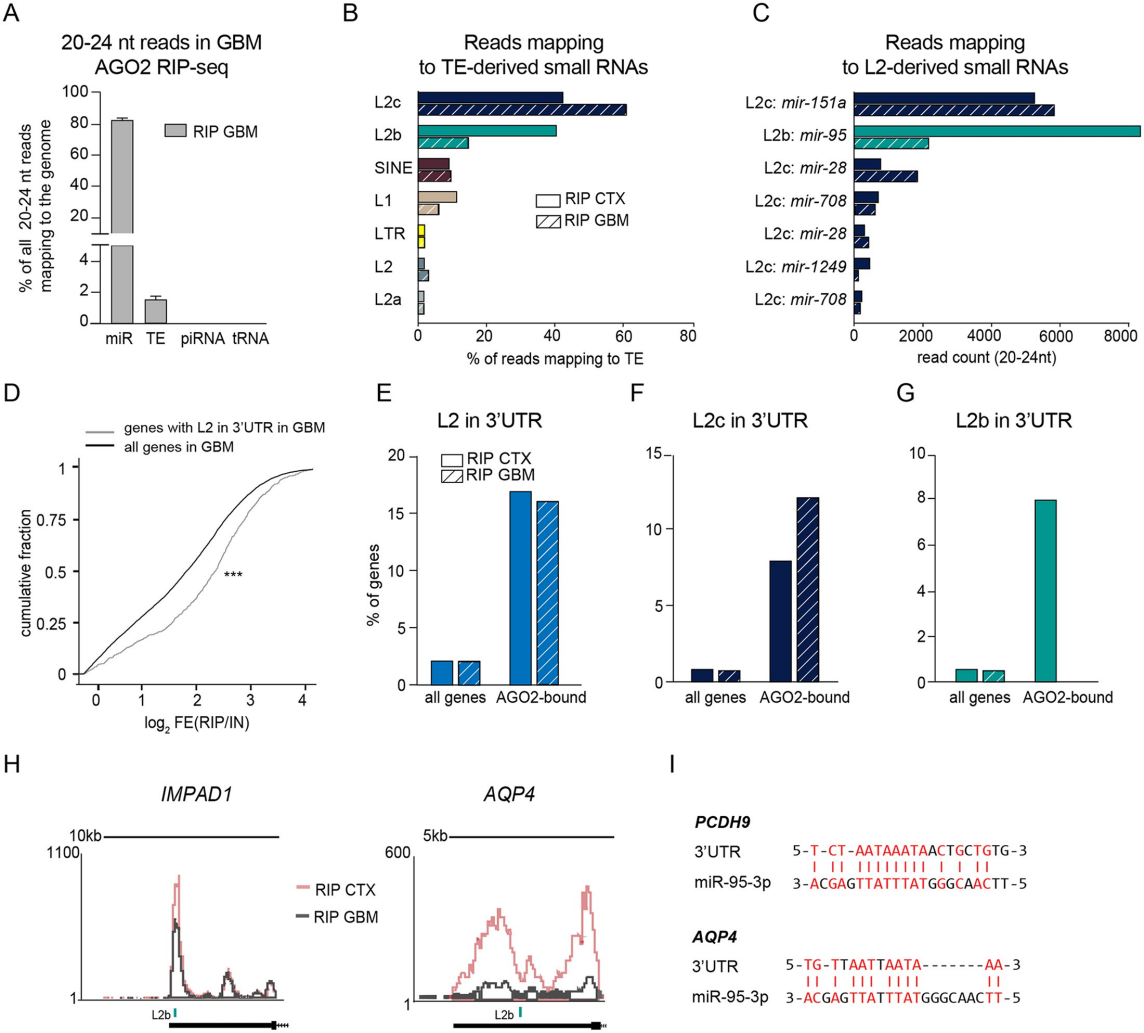
AGO2-associated L2-miRNAs and L2-carrying genes are altered in glioblastoma. A) Bar graph showing the percentage of 20–24 nt long read mapping to mature miRNAs (miR), transposable elements (TE), piwi RNAs (piRNAs) and transfer RNAs (tRNA). Data are represented as mean ± SEM (RIP GBM n = 6). B) Bar graph showing the percentage of uniquely aligned reads of 20–24 nt mapping to transposons in AGO2 RIP samples of cortex (CTX) and glioblastoma (GBM) tissue (RIP CTX n = 3; RIP GBM n = 6). C) Number of uniquely aligned reads mapping to L2 elements giving rise to precursor miRNAs (mir) in AGO2 RIP-seq samples from cortex and glioblastoma tissue (RIP CTX n = 3; RIP GBM n = 6). D) Cumulative fraction plot of fold changes of all genes and genes with L2 in their 3’UTR in RIP versus INPUT samples. ***p<0.001 Kolmogorov–Smirnov test. E-G) Bar plots showing the percentage of genes with L2 / L2b / L2c in the 3’UTR of all genes, and of the top 100 highest-expressed genes that are more than 4-fold enriched in RIP compared to input samples from cortex and glioblastoma tissue. H) Overlap of UCSC genome browser tracks showing examples of L2-carrying transcripts with altered AGO2 binding in glioblastoma compared to cortex. I) Potential L2b-derived target sites in *PCDH9* and *AQP4*. AGO2—Argonaute2, AGO2 RIP—AGO2 RNA immunoprecipitation, L1—LINE1, L2—LINE-2, LTR—long terminal repeat, SINE—short interspersed nuclear element.

Since miRNAs and TEs often display aberrant expression in human cancers [19, 20], we next investigated whether the expression of L2-miRNAs and their target network are altered in glioblastoma. We first conducted AGO2 RIP, followed by small RNA sequencing, on human brain biopsies obtained from glioblastoma patients (n = 6). We found that, as in cortex samples, AGO2-bound small RNAs in glioblastoma tissue displayed a high enrichment in reads of 20–24 nt while input samples had a broad RNA size profile (S3A Fig). When investigating the genomic origin of AGO2-bound small RNAs in glioblastoma, we found very similar results to the results from cortex tissue: most of the small RNAs were classical miRNAs, and there was only very limited evidence for AGO2 binding of tRFs or piRNA (Fig 6A).

We next analyzed the miRNA profile in glioblastoma and found miR-21 to be the most abundant miRNA in this tissue (S3B Fig). Differential expression analysis of miRNAs in glioblastoma samples compared to cortex samples showed altered expression of several miRNAs previously identified to be dysregulated in glioblastoma, for example miR-21, miR-10b, and miR-128 (S3C Fig) [20, 21]. We then analyzed TE-derived miRNAs and found that L2-miRNAs were also abundant in glioblastoma samples (Fig 6B). Interestingly, when analyzing individual L2-miRNAs, we found that the L2b-derived miR-95-3p appeared to be highly downregulated in glioblastoma samples (Fig 6C).

### AGO2 binding of L2-carrying genes is altered in human glioblastoma

To investigate how AGO2-bound genes carrying L2 in their 3’UTR are affected in glioblastoma we conducted AGO2 RIP, followed by total RNA sequencing. We found very similar results to cortex tissue when analyzing the global genomic distribution of reads. Input samples showed a high amount of rRNA, which was strongly decreased in the RIP fraction (S3D Fig). Moreover, reads mapping to 3’UTRs of genes and to TEs were enriched in the RIP samples of glioblastoma tissue compared to input fractions (S3D Fig).

We next focused our analysis on transcripts carrying L2 in their 3’UTR. Like in cortex samples, we saw an enrichment of genes carrying L2 fragments in the 3’UTR (Fig 6D). We also found that, while the overall numbers of the respective transcripts were very similar in glioblastoma and cortex samples (Fig 6E), there were profound differences when transcripts carrying L2b and L2c were analyzed separately (Fig 6F and 6G). Genes carrying L2c in their 3’UTR were enriched in the RIP samples from both sample types (Fig 6F). In contrast, we did not detect any AGO-bound L2b-carrying genes in glioblastoma tissue, although they were bound to AGO2 in cortex samples (Fig 6G and 6H). This is in line with the small RNA data, where the L2b-derived miR-95 was highly downregulated in glioblastoma (Fig 6C). Thus, this analysis directly links the altered expression of L2b-derived miR-95 to the lack of L2b-carrying targets bound to AGO2 in glioblastoma tissue. Interestingly, several of these L2b-targets, which are not bound by AGO2 in glioblastoma, are implicated in glioma, such as *PCDH9* [22] and *AQP4* [19] and show similar non-canonical target sites as we found in other L2-miRNA targets (Fig 6I).

## Discussion

In this study, we demonstrate that L2 elements as part of pri-miRNA transcripts serve as a source for several miRNAs that are bound by AGO2 proteins in the human brain with the ability to target hundreds of transcripts carrying L2 fragments in their 3’UTR. Our study therefore provides a model for how small RNAs derived from TEs play a role in post-transcriptional networks in the human brain. L2-miRNAs and targets are ubiquitously expressed across multiple human tissues, generally at low levels. This suggests that this network acts as a post-transcriptional buffer, providing additional robustness to the levels of housekeeping genes. This is in line with previous observations where L2-derived miRNAs, such as miR-95 and miR-151, were found to control basic cellular mechanisms such as lysosomal function and cell migration [11, 12].

L2 elements invaded the genome of our ancestors some 100–300 million years ago, well before mammalian radiation [17]. Throughout evolution, these TE sequences likely were subjected to a high evolutionary pressure, leading to the degradation of non-functional sequences: hence, only leaving fragments of sequences remaining in the genome. Today, a portion of these sequences form a post-transcriptional network composed of miRNAs and miRNA-target templates present in the 3’UTR of thousands of protein-coding genes. It is tempting to speculate that many miRNAs originally emerged from TEs, since TEs are scattered throughout the genome in high numbers and therefore have the potential to give rise to a large number of miRNAs and miRNA target sites. However, since most miRNAs are evolutionarily old (more than a billion years old) it is impossible to determine their origin with certainty. Several TEs have previously been implicated in both the generation of miRNAs as well as miRNA targets [9, 10, 14, 23, 24]. However, most observations concerning TE-derived miRNA-target interactions are based on computational predictions: experimental evidence for TE-derived miRNA-target interactions have been, up until now, limited. Key to the findings in this study is the use of AGO2 RIP on human tissue, coupled to next-generation sequencing on both small and total RNA. By using this approach, we could confirm and demonstrate the functionality of L2-miRNAs and show that transcripts with L2 in their 3’UTR are AGO2-bound miRNA targets.

It is commonly accepted that miRNAs regulate genes via the seed sequence, which spans from nucleotides 2–7 in the 5’ end of the miRNA [25]. However, several alternatives to canonical seed sequences have been proposed [6, 26]. For instance, miR-151 and miR-28 have previously been found to use centered seed pairing to regulate genes [6]. Since TE-derived miRNAs have highly similar sequences to TEs in the 3’UTR of genes, such miRNAs can most likely use extensive base-paring for target recognition and regulation. This suggests that non-canonical miRNA target sites might be more broadly used than previously thought.

Glioblastoma is the most aggressive and most common type of brain tumor in adults and is characterized by vast cellular and genetic heterogeneity. In the material used in this study, we found that the previously identified glioma-associated miRNAs such as miR-21 and miR-10b were dysregulated. In addition, we found that the L2b-derived miR-95 was highly expressed in the cortex, but not in glioblastoma, which is in line with previous reports demonstrating reduced altered levels of miR-95 in glioma [27, 28], although its exact role in glioma remains poorly understood. Together with our finding that L2b-carrying transcripts are not bound to AGO2 in glioblastoma tissue, this suggests that miR-95 could play an important role in the regulation of transcripts related to tumor progression or tumor defense in glioblastoma. Future studies analyzing the function of miR-95 in glioblastoma are therefore warranted.

In summary, this work demonstrates the existence of a TE-based post-transcriptional regulatory network that shapes the expression of hundreds of TE-carrying transcripts, and hence provides an additional mechanism for TEs to influence crucial gene networks in human tissues.

## Methods

### Ethics statement

The use of human brain tissue was approved by the local Ethical Committee in Lund (212/2007 for epilepsy and H15 642/2008 for glioblastoma) in accordance with the declaration of Helsinki. Prior to each surgery, written informed consent was obtained from all subjects.

### Human tissue

Fresh human adult neocortical and glioblastoma tissues were obtained during resective surgery in patients suffering from pharmacologically intractable epilepsy or glioblastoma, respectively. The tissue was snap-frozen immediately following removal.

### Mouse brain tissue

All animal-related procedures were approved and conducted in accordance with the Committee for Use of Laboratory Animals at Lund University. For AGO2 RIP-seq, the striata of mice were quickly dissected and immediately homogenized and lysed in ice-cold lysis buffer.

### AGO2 RIP-seq

Fresh or snap-frozen tissue was homogenized in ice-cold lysis buffer (10 mM HEPES (pH = 7.3), 100 mM KCl, 0.5% NP40, 5 mM MgCl_2_, 0.5 mM dithiothreitol, protease inhibitors, recombinant RNase inhibitors, 1 mM PMSF) using TissueLyser LT (30 Hz, 4 minutes).

Homogenates were centrifuged twice for 15 minutes at 16,200 × g, 4°C to clear the lysate. 1/10 of the sample was then saved as input (total RNA) control. The remaining lysate was incubated with anti-AGO2-coated Dynabeads Protein G beads (Life Technologies) at 4°C for 24 hours with end-over-end rotation (AGO2 antibody: anti-Ago2-3148 for human brain tissue [29] and Sigma-Aldrich 2E12-1C9 for mouse tissue). As control, the lysate was incubated with a control antibody not targeting AGO proteins (rabbit anti-GFP polyclonal antibody). After incubation, the beads were collected on a Dynamagnet (1 minute, on ice) and gently resuspended in low-salt NT2 buffer (50 mM Tris-HCl (pH = 7.5), 1 mM MgCl_2_, 150 mM NaCl, 0.5% NP40, 0.5 mM dithiothreitol, 1 mM PMSF, protease inhibitors, recombinant RNAse inhibitors). The beads were transferred into a new collection tube and washed once with low-salt NT2 buffer, followed by two washes with high-salt NT2 buffer (50 mM Tris-HCl (pH = 7.5), 1 mM MgCl_2_, 600 mM NaCl, 0.5% NP40, 0.5 mM DTT, protease inhibitors, 1 mM PMSF, recombinant RNAse inhibitors). After the last washing step, the RNA fraction was resuspended in QIAzol buffer, and RNA was isolated from RIP and input samples according to the miRNeasy micro kit (Qiagen).

### cDNA library preparation and sequencing of human AGO2 RIP samples

cDNA library preparation was conducted using the NEB small RNA library prep kit for small RNA sequencing and the NuGen Ovation RNAseq V2 kit, followed by the Ovation Ultralow V2 Library or Ovation Rapid Library Systems, for total RNA sequencing. Illumina high-throughput sequencing (HiSeq2500 SR 1 × 50 run and HiSeq3000 1 × 50) was applied to the samples (total number of reads for small RNA sequencing: 344508344; total number of reads for total RNA sequencing: 527413532) at the UCLA Microarray Core Facility.

### Analysis of the genomic distribution of small RNA sequencing reads from AGO2 RIP-seq

To analyze the genomic distribution of reads, the data were aligned to the human reference genome (hg38) using STAR (2.5.0a) [30], allowing multimapping and two mismatches per 22bp (--outFilterMismatchNoverLmax 0.05). Reads were quantified with the SubRead package FeatureCounts [31] (minimal overlap 19 nt) using annotations obtained from miRbase [32] to quantify mature miRNAs, the UCSC genome browser RepeatMasker track (GRCh38) to quantify repeats, tRNA annotations obtained from the UCSC table browser (GRCh38) to quantify tRNAs, and annotations from piRNAbank to quantify piRNAs [33].

### Analyses of TE-derived small RNAs bound to AGO2

To analyze small RNAs derived from individual TEs, the data were aligned to the human reference genome (hg38) using STAR (2.5.0a), allowing for 0 mismatches. Reads that mapped equally well to more than one locus were discarded (--outFilterMultimapMax 1), while reads with a better alignment score for one locus than for any other position were kept (--outFilterMultimapScoreRange 1). These parameters enable the specific, expressed locus to be quantified, while reads with repeated identical sequences that cannot be assigned to any specific locus are discarded. As control, multimapping reads were kept, although there were no significant effects on the quantification and detection of TE-derived small RNAs, indicating that there are no or very few identical TE-derived small RNAs.

To detect TE-derived miRNAs, only reads with a length of 20–24 nt were included in the analysis. Furthermore, to identify high-confidence miRNAs, 3p and 5p strands and typical Dicer cleavage patterns had to be present. We also assessed conservation of the small RNAs by using the 100 vertebrates Basewise Conservation data by phyloP from the PHAST package.

To align the L2c-derived small RNAs to the L2c consensus, we used the raw alignment of the L2c consensus to the reference genome (GRCh38) generated by RepeatMasker to annotate genomic L2c. This alignment can be assessed through the detailed visualization of RepeatMasker annotations in the UCSC genome browser. The genomic-to-L2c consensus alignment by RepeatMasker was used to map the TE-derived small RNA to its relative location in the L2c consensus.

### Expression analysis of L2-small RNAs in different tissues

To analyze the expression of L2-small RNAs, we downloaded expression profile data of miRNAs in 400 human samples from http://fantom.gsc.riken.jp/5/suppl/De_Rie_et_al_2017/ (file: human.srna.cpm.txt) [15] and extracted the counts per million (cpm) data for the 19 L2-miRNAs we identified in this study (Table 1). A pseudo-count of 1.0 was added and the logarithm (base 2) was taken for the values. Hierarchical clustering was done using Euclidean distance with single linkage.

### Conservation analysis of L2-derived miRNAs

Sequences and coordinates of mature miRNAs were obtained from miRbase [32] and coordinate conservation was analyzed using genome browser annotations.

### Expression analysis of miRNA hairpins in DGCR8 and Dicer knockout mouse embryonic stem cells

In order to analyze the dependence of L2-derived miRNAs on Dicer and DGCR8, we extracted fold changes and read numbers from a previously published small RNA sequencing data set [16].

### Expression analysis of genes carrying L2 in 3’UTR

To obtain a list of transcripts with L2 elements in their 3’UTRs, Ensembl 3’UTR annotations were overlapped with L2 RepeatMasker annotations using BEDTools intersection requiring at least 1 bp overlap. A publicly available dataset consisting of transcriptome levels of 27 different tissue types across 95 individuals was downloaded from www.ebi.ac.uk/arrayexpress/ (accession ID E-MTAB-1733) [18]. FPKM values for 1932 genes out of the 2042 genes previously identified as having L2 elements in their 3’UTRs were extracted from the above data. These genes were grouped into seven different classes depending on their tissue-specificity characteristics as described in Fagerberg et al. [18]. A pseudo-count of 1.0 was added and logarithm (base 2) was computed before performing hierarchical clustering (Euclidean distance and single linkage).

### Analysis of structural conservation of L2 elements in the human genome

To get the coverage of bases derived from each base position in L2c consensus, the relative position of genomic L2c elements aligning to L2c consensus were extracted from the RepeatMasker output file (http://www.repeatmasker.org/genomes/hg38/RepeatMasker-rm405-db20140131/hg38.fa.out.gz). The coverage module of BEDTools (v2.24.0) was used to calculate the consensus coverage, which shows how many of the genomic L2c copies have the given position in the consensus sequence.

### Analyses of total RNA sequencing data from AGO RIP on human tissue

For the expression analyses of AGO2-bound genes, reads were aligned to the human reference genome (hg38) using STAR (2.5.0a) with default settings. 3’UTR annotations were obtained from Ensembl [34].

### Analyses of total RNA sequencing data to identify L2-carrying transcripts

To identify L2 elements within 3’UTRs, reads were aligned to the human reference genome (hg38) using STAR (2.5.0a), allowing for two mismatches per 50 nt (--outFilterMismatchNoverLmax 0.04), and discarding multi-mapping reads. The files obtained were intersected with L2 annotations, obtained from RepeatMasker with the requirement of 80% overlap (-f 0.8). The reads were then intersected with 3’UTR annotations (Ensembl) with a requirement of at least 1 bp overlap. The intersection was conducted using the BEDTools (2.26.0) intersect module.

### Identification of miRNA target sites in L2 elements in the 3’UTR of genes

The L2 sequence in the 3’UTR of a gene was aligned to the sequence of an L2-miRNA using the EMBOSS Needle nucleotide pairwise alignment tool [35] to find sequence complementarity. The following parameters were used: DNAfull substitution scoring matrix, 10-gap open penalty and 0.5-gap extend penalty, no end-gap penalty applied.

### Luciferase reporter assay

A 400 bp sequence incorporating the L2-derived miRNA binding sites in the 3’UTR of *CLIP3*, *IMPAD1*, *YWHAZ*, *TPP1*, and *ADD2* was cloned into the dual luciferase reporter vector pSICHECK-2 (Promega). The luciferase reporter constructs were co-transfected with either a GFP overexpression vector, a miR-10a non-targeting over-expression construct or the respective L2-derived miRNA overexpression vector into three independent replicates of 293T cells using Turbofect (Fermentas). 48 hours after transfection, cells were assayed for luminescence using a dual-luciferase assay (Promega). One-way ANOVA followed by a Tukey’s multiple comparison post-hoc test were performed in order to test for statistical significance. Data are presented as mean ± SEM.

### Data access

All RNA sequencing data have been submitted to the NCBI Gene Expression Omnibus database and assigned the GEO series accession number GSE106810.

## Supporting information

S1 Fig Related to Fig 1. A) Read size distribution of cortex (CTX) RIP and input samples. B) Bar graph showing the percentage of 20–24 nt long reads in cortex tissue mapping to mature miRNAs (miR), transposable elements (TE), piwi RNAs (piRNA) and transfer RNAs (tRNA). Data is represented as mean ± SEM (RIP CTX n = 3). C) Pie chart showing the percentage of reads mapping to individual miRNAs. D) Schematics of a full-length (FL) L2 element (red box indicates the 3’ end) and alignment of L2c-miRNAs to the 3’ end (position 3222–3387) of the L2 consensus sequence. AGO—Argonaute.
(TIF)

S2 Fig Related to Fig 3. A) Examples of Fantom5 CAGE data for mir-151a and mir-493 [15].
(TIF)

S3 Fig Related to Fig 5. A) Read size distribution of glioblastoma RIP and input samples. B) Pie chart showing the percentage of reads mapping to individual miRNAs in glioblastoma tissue. C) Dot plot depicting miRNA expression in glioblastoma RIP compared to cortex RIP samples. The log_2_ transformed base Mean is plotted against the log_2_ transformed fold change. BH-adjusted p value < 0.05. D) Genomic distribution of total RNA sequencing reads in AGO2 RIP and input samples (GBM, n = 5). Data is presented as mean ± SEM.
(TIF)
