# Supplementary figures and images for: LINE-2 transposable elements are a source of functional human microRNAs and target sites
Source: PLoS Genet. 2019 Mar 13;15(3):e1008036. doi: 10.1371/journal.pgen.1008036 (PMC6433296; doi:10.1371/journal.pgen.1008036)

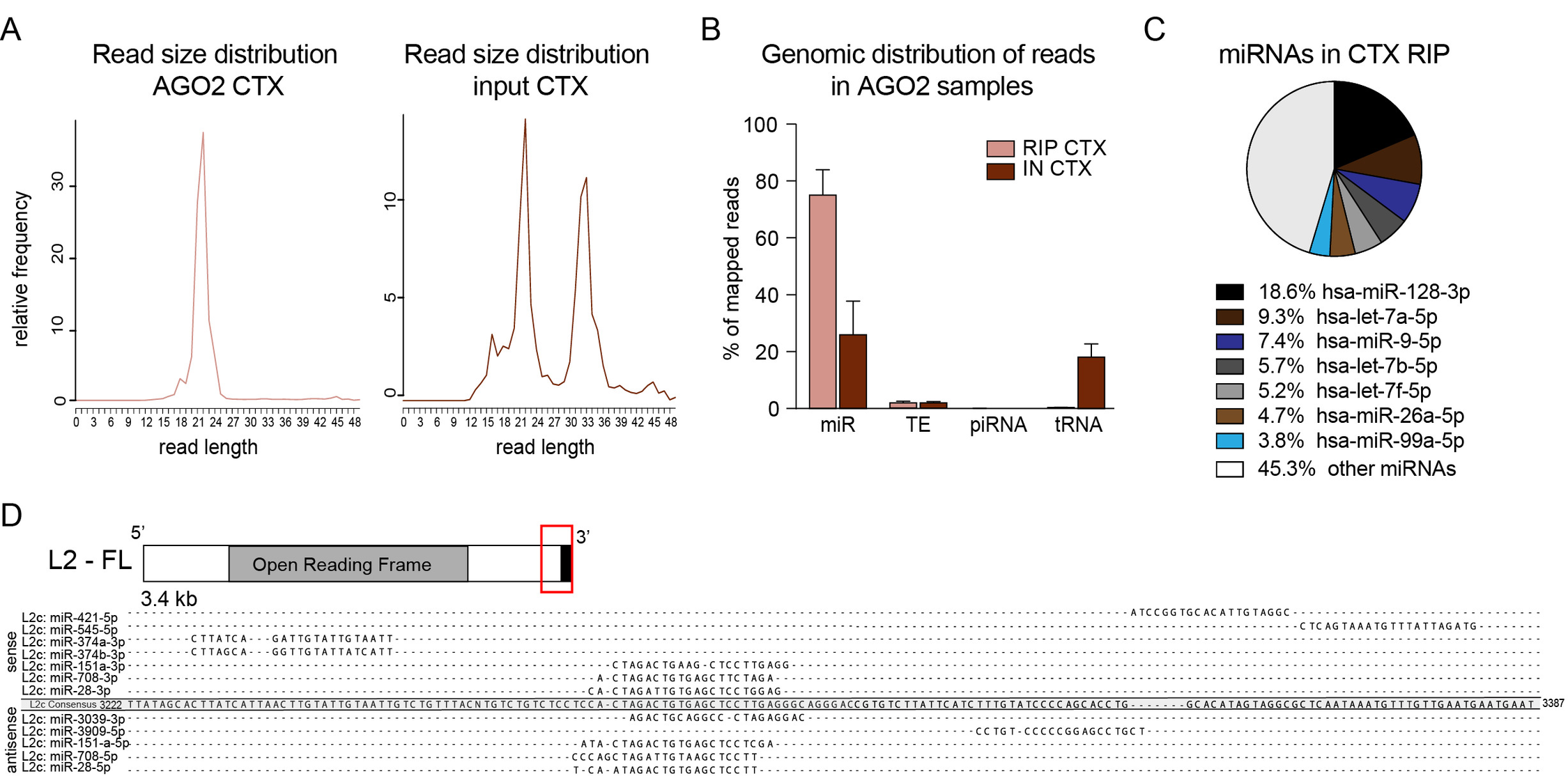

Supplement: S1 Fig — A) Read size distribution of cortex (CTX) RIP and input samples. B) Bar graph showing the percentage of 20–24 nt long reads in cortex tissue mapping to mature miRNAs (miR), transposable elements (TE), piwi RNAs (piRNA) and transfer RNAs (tRNA). Data is represented as mean ± SEM (RIP CTX n = 3). C) Pie chart showing the percentage of reads mapping to individual miRNAs. D) Schematics of a full-length (FL) L2 element (red box indicates the 3’ end) and alignment of L2c-miRNAs to the 3’ end (position 3222–3387) of the L2 consensus sequence. AGO—Argonaute. (TIF) [file pgen.1008036.s001.tif]

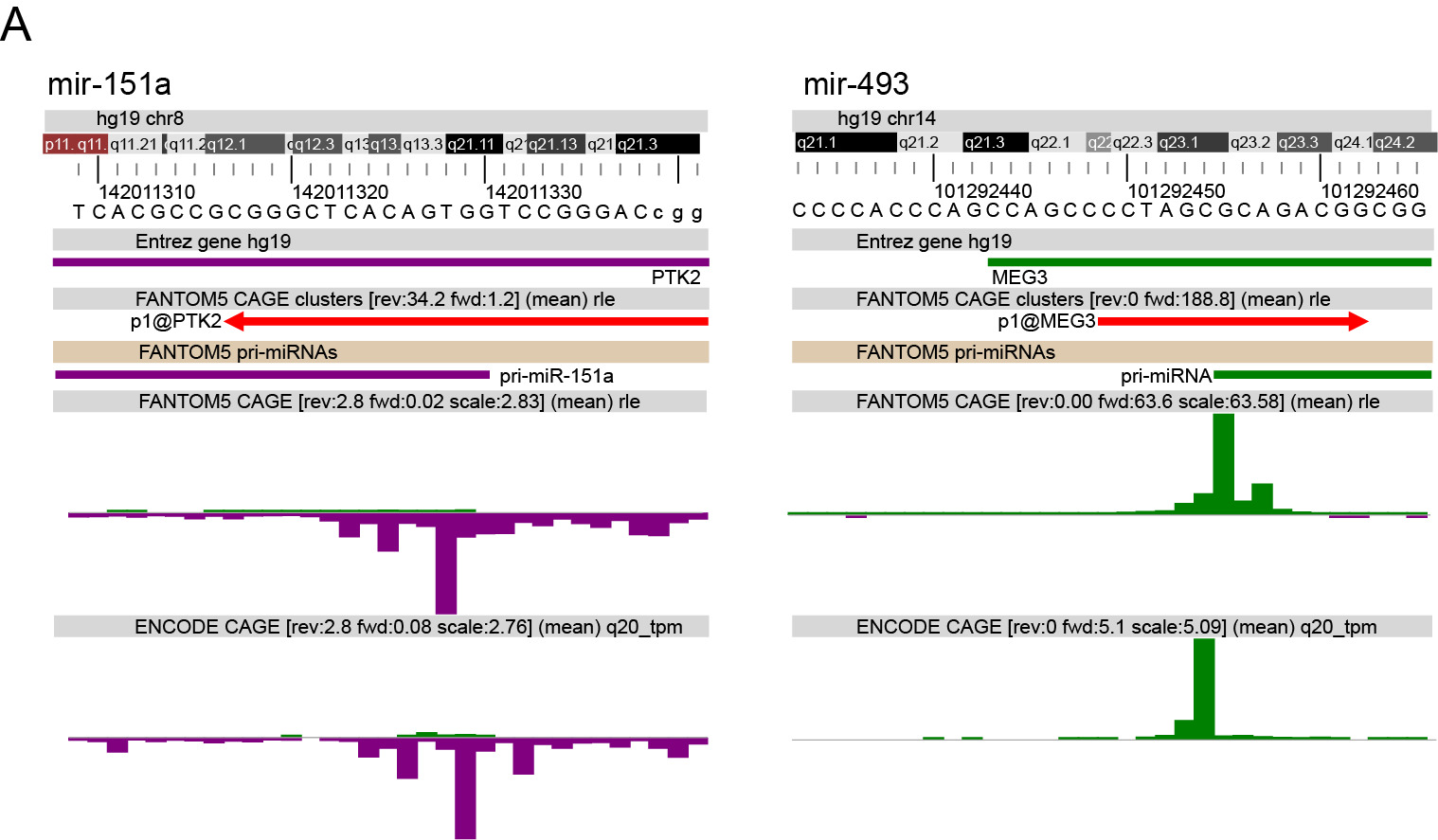

Supplement: S2 Fig — A) Examples of Fantom5 CAGE data for mir-151a and mir-493 [15]. (TIF) [file pgen.1008036.s002.tif]

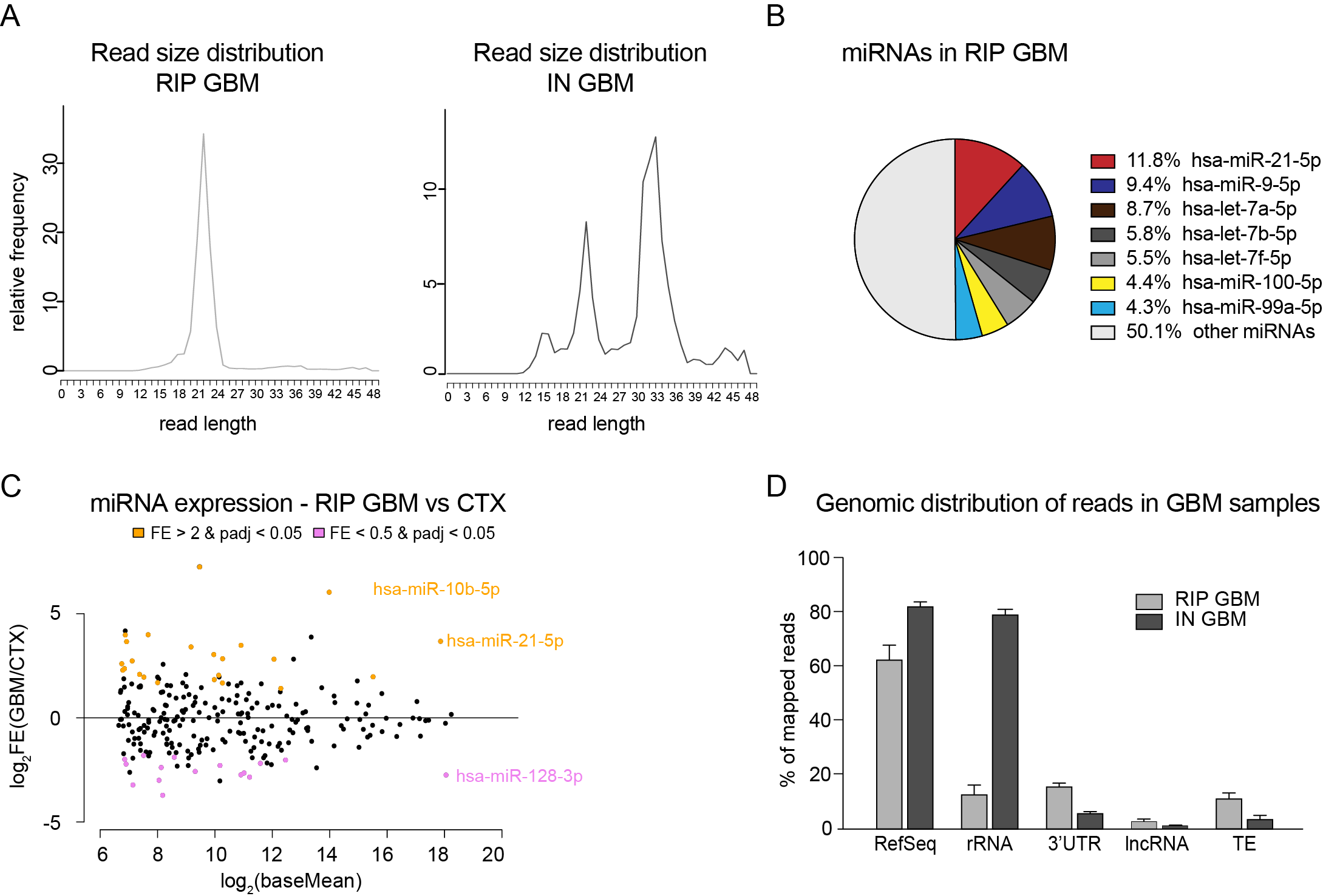

Supplement: S3 Fig — A) Read size distribution of glioblastoma RIP and input samples. B) Pie chart showing the percentage of reads mapping to individual miRNAs in glioblastoma tissue. C) Dot plot depicting miRNA expression in glioblastoma RIP compared to cortex RIP samples. The log2 transformed base Mean is plotted against the log2 transformed fold change. BH-adjusted p value < 0.05. D) Genomic distribution of total RNA sequencing reads in AGO2 RIP and input samples (GBM, n = 5). Data is presented as mean ± SEM. (TIF) [file pgen.1008036.s003.tif]
